# Smell and taste in cervical dystonia

**DOI:** 10.1007/s00702-020-02156-4

**Published:** 2020-02-15

**Authors:** Thorsten Herr, Thomas Hummel, Marcus Vollmer, Carsten Willert, Birgitt Veit, Julie Gamain, Robert Fleischmann, Bernhard Lehnert, Jan-Uwe Mueller, Andrea Stenner, Martin Kronenbuerger

**Affiliations:** 1 Department of Neurology, University Medicine Greifswald, Ferdinand-Sauerbruch-Strasse, 17475 Greifswald, Germany; 2 Department of Otorhinolaryngology, TU Dresden, Dresden, Germany; 3 Institute of Bioinformatics, University Medicine Greifswald, Greifswald, Germany; 4 Neurology Group Practice, Stralsund, Germany; 5 Neurology Group Practice, Neubrandenburg, Germany; 6 Department of Otorhinolaryngology, University Medicine Greifswald, Greifswald, Germany; 7 Department of Neurosurgery, University Medicine Greifswald, Greifswald, Germany; 8 Outpatient Department of Neurology, Paracelsus Clinic Zwickau, Zwickau, Germany; 9 Department of Neurology, Johns Hopkins University, Baltimore, MD USA

**Keywords:** Olfaction, Gustatory functioning, Network disorder, Cerebellum, Basal ganglia, Sensorimotor cortex

## Abstract

The pathophysiology of cervical dystonia is not completely understood. Current concepts of the pathophysiology propose that it is a network disorder involving the basal ganglia, cerebellum and sensorimotor cortex. These structures are primarily concerned with sensorimotor control but are also involved in non-motor functioning such as the processing of information related to the chemical senses. This overlap lets us hypothesize a link between cervical dystonia and altered sense of smell and taste. To prove this hypothesis and to contribute to the better understanding of cervical dystonia, we assessed olfactory and gustatory functioning in 40 adults with idiopathic cervical dystonia and 40 healthy controls. The Sniffin Sticks were used to assess odor threshold, discrimination and identification. Furthermore, the Taste Strips were applied to assess the combined taste score. Motor and non-motor deficits of cervical dystonia including neuropsychological and psychiatric alterations were assessed as cofactors for regression analyses. We found that cervical dystonia subjects had lower scores than healthy controls for odor threshold (5.8 ± 2.4 versus 8.0 ± 3.2; *p* = 0.001), odor identification (11.7 ± 2.3 versus 13.1 ± 1.3; *p* = 0.001) and the combined taste score (9.5 ± 2.2 versus 11.7 ± 2.7; *p* < 0.001), while no difference was found in odor discrimination (12.0 ± 2.5 versus 12.9 ± 1.8; *p* = 0.097). Regression analysis suggests that age is the main predictor for olfactory decline in subjects with cervical dystonia. Moreover, performance in the Montreal Cognitive Assessment is a predictor for gustatory decline in cervical dystonia subjects. Findings propose that cervical dystonia is associated with diminished olfactory and gustatory functioning.

## Introduction

Cervical dystonia (CD) is the most common form of adult onset focal dystonia (Albanese et al. 2013; ESDE 2000). Beside dystonia affecting the head, neck and shoulder muscles as well as action induced tremors, there are numerous non-motor deficits in CD (Kuyper et al. 2011). These include sensory abnormalities, such as pain (Tinazzi et al. 2019), sensory tricks (Sarasso et al. 2020), altered temporal and spatial discrimination (Junker et al. 2019; Conte et al. 2019) and impaired temperature detection threshold (Paracka et al. 2017). Furthermore, there are mild neuropsychological deficits (such as deficits of memory, verbal fluency and executive functioning) as well as psychiatric alterations (such as depression and anxiety) (Kuyper et al. 2011).

The pathophysiology of CD is not completely understood. Current concepts of its pathophysiology propose alterations in a network including the sensorimotor cortex, basal ganglia and cerebellum (Prudente et al. 2014; Quartarone and Hallett 2013; Shakkottai et al. 2017). While this network is primarily concerned with sensorimotor control (Quartarone and Hallett 2013), the basal ganglia and cerebellum are also important for non-motor functioning (Alexander et al. 1986; Bodranghien et al. 2016; Bostan et al. 2013; Gerwig et al. 2010; Eisinger et al. 2018; Saint-Cyr 2003; Strick et al. 2009; Weintraub and Zaghloul 2013). Additionally, there is evidence for the involvement of basal ganglia and cerebellum in the chemical senses. Tracing studies in animals revealed that basal ganglia receive input from both the primary gustatory cortex (which is primarily the insular cortex) (Fudge et al. 2005) and primary olfactory cortex (which includes olfactory tubercle) (Alexander et al. 1986; Eslinger et al. 1982; Russchen et al. 1985), whereas the cerebellum receives olfactory input via the ventral tegmental area from the piriform cortex (Ikai et al. 1992, 1994). Additionally, functional imaging studies in healthy human subjects showed activation of the cerebellum and the basal ganglia with olfactory tasks (Savic 2002). Furthermore, focal and degenerative cerebellar disease causes olfactory decline (Connelly et al. 2003; Mainland et al. 2005; Zobel et al. 2010). The sensorimotor cortex receives afferent projections from the central gustatory tracts (Heckmann et al. 2003; Mascioli et al. 2015). In addition, functional imaging showed activation of the sensorimotor cortex with gustatory stimulation of the tongue (Mascioli et al. 2015; Wistehube et al. 2018).

Based on the overlap between neuroanatomical regions involved in the pathophysiology of CD and processing of information related to the chemical senses, altered sense of smell and taste may be found in CD. In fact, few studies have explored the sense of smell in dystonia. Compared to a group of healthy controls, odor identification score as assessed by the University of Pennsylvania Smell Identification Test (UPSIT) in 14 dystonia subjects was slightly lower than in healthy controls, but the difference was not statistically significant (Silveira-Moriyama et al. 2009). In a different study, a statistically lower odor identification score as assessed by the UPSIT was found in five dystonia subjects with mutations in the GNAL gene of only one family (Vemula et al. 2013). More recently, odor identification, odor discrimination and odor threshold were assessed by the Sniffin Sticks (Hummel et al. 1997) in 58 subjects with CD (Marek et al. 2018). Results were compared to data of healthy controls matched only for age and gender from a database. Subjects with CD had lower scores for odor threshold and odor identification, while their score for odor discrimination was in the normal range.

There are several confounding factors that have not or have been only partially taken into account in past studies of olfaction in CD. Sinusoidal disorders can cause olfactory decline (Hummel et al. 2016). Epidemiological factors such as age and gender effect olfaction, but also education and tobacco smoking (Vennemann et al. 2008). Additionally, cognitive domains including executive functioning, memory and verbal fluency impact performance on olfactory tests (Hedner et al. 2010; Westervelt et al. 2005). Psychiatric alterations including depression and anxiety have also been found to impede the sense of smell (Croy and Hummel 2017; Kamath et al. 2018). In terms of olfaction in CD, these factors are possibly important as cognitive and psychiatric alterations are non-motor features of CD (Kuyper et al. 2011).

Little is known about the sense of taste in movement disorder subjects (Cecchini et al. 2015; Lang et al. 2006; Shah et al. 2009). To the best of our knowledge, taste has not been assessed in CD so far. This is surprising as, similar to the sense of smell, the sense of taste contributes to quality of life (Croy et al. 2014; Doty 2019).

To contribute to the better understanding of CD, the primary aim of this study was to systematically examine olfactory and gustatory functioning in CD subjects compared to healthy controls matched for epidemiological factors such as age, sex and education. Diseases which could impede the chemical senses such as sinusoidal disorders were exclusion criteria through history and clinical examination. Also, clinical characteristics of dystonia, cognitive functioning and psychiatric alterations were analyzed as cofactors of olfactory and gustatory performance.

## Methods

### Study participants

CD subjects were recruited through the Movement Disorder Center of the Department of Neurology at the University Hospital Greifswald. Inclusion criteria for CD subjects were adult age onset idiopathic cervical dystonia consistent with to established criteria (Albanese et al. 2013; ESDE 2000). Excluding criteria were any central nervous system pathologies other than CD, history of or current radiation and chemotherapy, use of central nervous active medication, anatomical deformities or pathologies of mouth, ear and nose or any medical or surgical condition which could impede smell or taste (Heckmann et al. 2003; Landis et al. 2011; Patel and Pinto 2014), a score below 26 in the Montreal Cognitive Assessment (MoCA) (Freitas et al. 2012) as well as history of head trauma and abnormal findings on neuroimaging studies or laboratory work-up as done for routine care. CD subjects treated with botulinum toxin (BTX) were examined at least 3 months after their last BTX treatment. Healthy control subjects were selected to match age, sex, handedness and education of CD subjects. The same exclusion criteria for the selection of CD subjects were applied for the selection of healthy controls. For any CD subject who smoked tobacco, a control subject with a similar smoking burden (number of cigarettes consumed per day) was included.

### Clinical interview, examination and scores

Demographic as well as clinical data such as onset of dystonia and family history of a movement disorder were collected during an in-person interview in addition to the review of the clinical charts. The test battery applied in all study participants included the MoCA to assess overall cognitive functioning (Freitas et al. 2012) the Trail-Making-Test (TMT) for the assessment of executive functioning (Brown et al. 1958), the Digit Span Test (DST) (De Paula et al. 2016) to assess short-term memory, the FAS-Test to assess verbal fluency (Machado et al. 2009) and the Brief Symptom Inventory (BSI) (Franke 2000) to assess depression and anxiety. A neurological exam in all study subjects was done by a fellowship-trained, senior movement disorders neurologist (MK). The complete Toronto Western Spasmodic Torticollis Rating Scale (TWSTRS) (Consky and Lang 1994) was done in all CD subjects in this study. Tremor was assessed using part one of the Clinical Rating Scale for Tremor (Fahn et al. 1988). When tremor in CD affected the head, the tremor was classified as dystonic tremor (DT), whereas when the tremor affected a different body part, such as the arms, it was classified as tremor associated with dystonia (TAWD) (Bhatia et al. 2018). The nose was inspected by anterior rhinoscopy and the oral cavity was inspected by the use of a tongue depressor and an examination light to rule out other causes of olfactory or gustatory decline (Hummel et al. 2016). All study participants were asked to rate their overall olfactory and gustatory functioning on a scale from zero (absence) to ten (excellent), with five representing an average olfactory and gustatory functioning. The participants were requested not to drink, eat or smoke one hour before the beginning of the olfactory and gustatory examination. All study participants were examined in a well-rested state.

### Olfactory testing

The Sniffin Sticks were used to assess olfactory functioning including odor threshold, odor identification and odor discrimination (Hummel et al. 1997). Sniffin Sticks are felt-tip pen-like dispensers that contain odorants. For olfactory testing, the cap was removed and the stick was presented under the nostrils for 3 s. During the entire olfactory assessment, all participants were blindfolded to prevent visual identification.

For odor threshold testing, 16 triplets were used. Each triplet contained one stick with the odorant *n*-butanol and two sticks with a solvent. The three sticks of a triplet were presented to the study participants in a randomized order. In a three alternative forced choice procedure (3-AFC procedure) they were asked to identify the odor-containing pen. The assessment started with the lowest concentration. If the subjects gave a wrong answer, the triple with the next higher odor concentration was presented. When the stick containing the odor was correctly identified, the same odor concentration was presented again. After two successful trials the next lower concentration was presented. Incorrect answers led to the presentation of the next higher concentration. The reversals between lower to higher or higher to lower concentration were considered inflection points. The odor threshold was defined as the mean concentration of the last 4 of 7 inflection points. To prevent habituation and fatigue, a 30-s break was done between every triplet. Odor discrimination was assessed using 16 triplets with two sticks containing the same odorant and one stick with a different odorant. In a 3-AFC procedure the participants had to choose which pen smelled different to the other two. All triplets were presented in a randomized order and a break of 30 s between every triplet was given. To assess odor identification capability, 16 common odors were presented. The participants could choose one out of four answer options and a 30-3s break was taken between the presentation of the odors. The score range was 0 (poor result) to 16 (excellent result) for each of the three olfactory tests. The sum of the threshold, discrimination, and identification test was added to the composite olfactory score (maximum score 48). A composite olfactory odor score below 30 was defined as hyposmia (Haehner et al. 2009).

### Gustatory assessment

The sense of taste was assessed using the Taste Strips (Mueller et al. 2003). These filter paper strips were impregnated with four different taste qualities (sweet, sour, salty and bitter). Every taste was represented in four concentrations, beginning with the lowest. The taste strips were placed on the anterior part of the tongue. The participants were allowed to take a sip of water between every taste strip presented. In total, 18 strips were presented. Among them were two blanks without a taste quality. All strips were presented in increasing concentration and taste qualities were tested in a predetermined pseudo-randomized order. Every time a strip was presented, the participants had to choose an answer from the following options: “sweet”, “sour”, “salty”, “bitter” or “no taste”. The scores of the four taste qualities tested were added to the composite taste score. The results of the blanks were not counted. The score range was 0 (poor result) to 16 (excellent result). A composite taste score below nine was defined as a hypogeusia (Mueller et al. 2003).

### Statistics

Statistical analysis was performed using SPSS Statistics 25 software (SPSS Inc., Chicago, IL, USA). The test of Kolmogorov–Smirnov indicated that the data were normally distributed. Independent samples Student *t* test (two-tailed) was used to compare results of the CD subjects to healthy controls. The chi-square test was applied to compare prevalences. In case of statistically significant differences between CD subjects and healthy controls in the tests for the chemical senses, multiple linear regression analysis in the stepwise mode was done to assess whether clinical characteristics of CD predicted performance of the olfactory or gustatory tests. Factors included in these analyses were age, sex, tobacco smoking, education, disease duration, family history of CD, BTX treatment, TWSTRS part one to three, DT and TAWD as assessed with the CRST part one, performance in the cognitive tests as well as depression and anxiety sub-scores of BSI.

## Results

### Study participants

Details on the characteristics of the study participants can be found in Table 1. All participants were Caucasian and righthanded. DT was found in 25 of the 40 CD subjects and 12 of these CD subjects with DT had additionally TAWD. Besides the clinical signs and symptoms of CD in the CD subjects, the clinical neurological exam and exam of the nose and mouth were normal in all study participants. There were eight CD subjects with a family member who had CD as well. Thirty CD subjects had obtained BTX in the past, while ten CD subjects never had BTX before.

**Table 1:** Demographic and clinical data of the study participants

	CD subjects	Healthy controls	*p* value
*n*	40	40	1
Age, years	61.8 ± 10.9	61.6 ± 12,2	0.962
Gender (female/male)	23/17	23/17	1*
Education, years	10.0 ± 1.4	10.3 ± 1.7	0.321
Smokers	9	9	1*
Smoking burden in smokers	16.0 ± 4.7	17.0 ± 5.1	0.826
MoCA	28.6 ± 1.4	29.0 ± 1.3	0.287
ΔTMT, seconds	84.2 ± 66.6	50.0 ± 28.7	0.004
DST	15.5 ± 3.4	16.0 ± 3.4	0.511
FAS	32.1 ± 10.3	37.7 ± 14.8	0.052
BSI depression-subscore	2.8 ± 3.8	0.8 ± 1.2	0.002
BSI anxiety-subscore	5.1 ± 4.3	1.9 ± 1.8	< 0.001
Family history of CD, *n*	8	–	–
Disease duration, years	13.1 ± 10.3	–	–-
TWSTRS part A	16.2 ± 6.1	–	–
TWSTRS part B	8.9 ± 8.8	–	–
TWSTRS part C	6.8 ± 4.9	–	–

Values are mean ± standard deviation. *p* values marked with * are based on chi-square test, all other *p* values are based on unpaired *t* test

*CD* cervical dystonia, *n* number of participants, *Smoking burden in smokers* number of cigarettes consumed per day, *MoCA* Montreal Cognitive Assessment (Freitas et al. 2012), *ΔTMT* difference between Trail-Making-Test part B—part A (Brown et al. 1958), *DST* sum of Digit-Span-Test (De Paula et al. 2016), *FAS* FAS-Test (Machado et al. 2009), *BSI* brief symptom inventory (Franke 2000), *TWSTRS* Toronto Western Spasmodic Torticollis Rating Scale (Consky and Lang 1994)

### Olfactory ability

The group of CD subjects had lower scores than the group of healthy controls in the composite olfactory score (Table 2), the odor threshold test and the odor identification test, while no statistically significant difference was found in the odor discrimination test (Table 2). There were more CD subjects than healthy controls with hyposmia (52.5% versus 22.5%, *p* = 0.003). Subjective olfactory functioning in CD subjects was lower than in healthy controls (5.5 ± 1.9 versus 6.4 ± 1.8, *p* = 0.04).

**Table 2:** Olfactory and gustatory functioning in cervical dystonia subjects and healthy controls

	CD subjects	Healthy controls	*P* value
Composite olfactory score	29.5 ± 5.7	33.9 ± 4.9	< 0.001
Odor threshold	5.8 ± 2.4	8.0 ± 3.2	0.001
Odor discrimination	12.0 ± 2.5	12.9 ± 1.8	0.097
Odor identification	11.7 ± 2.3	13.1 ± 1.3	0.001
Composite taste score	9.5 ± 2.2	11.7 ± 2.7	< 0.001

Values are mean ± SD

*CD* cervical dystonia, *Composite olfactory score* sum of odor threshold, odor discrimination and odor identification of the Sniffin Sticks test (Hummel et al. 1997), *Composite taste score* result of the Taste Strips test (Mueller et al. 2003), p-values as assessed with unpaired *t* test

### Gustatory ability

The combined taste score was lower in CD subjects than in healthy controls (Table 2). There were more CD subjects than healthy controls with hypogeusia (35.0% versus 12.5%, *p* = 0.03). Subjective gustatory function in CD subjects was not statistically different from healthy controls (5.9 ± 1.8 versus 6.4 ± 1.4, *p* = 0.1).

### Clinical characteristics and chemical senses in CD

Multiple linear regression revealed a significant regression coefficient for age to predict low performance in the composite odor score ((*F*(1,38) = − 2.4, *p* = 0.02), *R*2 = 0.13, standard coefficient beta = − 0.36) and odor identification ((*F*(1,38) = − 2.7, *p* = 0.01), *R*2 = 0.16, standard coefficient beta = − 0.395). Additionally, multiple linear regression showed a significant regression coefficient for lower MoCA score to predict lower performance in the composite taste score in CD subjects ((*F*(1,38) = − 2.25, *p* = 0.03), *R*2 = 0.12, standard coefficient beta = − 0.34) and for TWSTRS—part 3 to predict lower performance in the odor threshold test ((*F*(1,38) = −  2.4, *p* = 0.02), *R*2 = 0.13, standard coefficient beta = − 0.36). All other regression equations did not reach the level of significance (all *p* values > 0.05).

## Discussion

This study systematically investigated the sense of smell and taste in 40 adult subjects with idiopathic CD compared with 40 healthy controls matched for age, sex, education and tobacco use. The main findings are that CD subjects had lower odor threshold, lower odor identification and lower composite taste scores than healthy controls.

Details on the neural circuits involved in odor threshold, odor discrimination and odor identification may help to explain findings. Peripheral olfactory structures such as the olfactory neuroepithelium and nerve are important for odor threshold (Hummel et al. 2016). For instance, diminished odor threshold is typically found in sinusoidal disorders (Patel and Pinto 2014; Whitcroft et al. 2017). While we excluded subjects with sinusoidal disorders, BTX can cause dry mouth (Dalton 2004; Dressler and Benecke 2003) and possibly dry nose, which could impede odor threshold. Although we cannot exclude this possibility, we believe it is less likely. The CD subjects on BTX treatment were assessed 3 months after the last BTX injections when the effects of BTX had worn off. Diminished odor threshold can be found with impairment of an olfacto-motor loop (Mainland et al. 2005). Within this circuit, the cerebellum as a structure for sensorimotor control regulates sniff volume inversely proportional to odor concentration in order to optimize sampling of sensory information (Bower 1997; Mainland et al. 2005; Sobel 1998; Zobel et al. 2010). We do not know if this is the case for CD, as we did not measure nasal air flow during the olfactroy tests. The use of a fixed-sniff method (Mainland et al. 2005) in future studies may reveal whether alterations of this loop contributes to diminished odor threshold in CD.

Functional imaging (Positron Emission Tomography and Magnet Resonance Tomography) in healthy subjects was applied to explore neural circuits involved in higher olfactory functions (Savic et al. 2000; Suzuki et al. 2001; Kareken et al. 2003; Kjelvik et al. 2012). Varying paradigms were used in the different studies, which limits comparability of findings. However, it appears that the olfactory system is organized in both a parallel and hierarchal manner (Savic et al. 2000). For example, the olfactory core regions including the right amygdala-piriform cortex, the right orbito-frontal and the prefrontal cortex, the left insular, the cingulum and the right thalamus were mutually activated by passive smelling of odors (= parallel organization) (Savic et al. 2000; Suzuki et al. 2001; Kareken et al. 2003; Kjelvik et al. 2012). In contrast, with increased complexity of the olfactory task, the activated areas were more and more remotely connected with the olfactory core regions (= hierarchical organization) (Savic et al. 2000). For instance, in contrast to passive smelling of an odor, tasks for odor threshold, odor discrimination and odor identification activated the right cerebellum (Savic et al. 2000). Moreover, some regions were only activated with certain tasks (for example, odor discrimination tasks activated the hippocampus (Savic et al. 2000; Kareken et al. 2003) and the caudate nucleus of the basal ganglia (Savic et al. 2000), while odor identification tasks activated the left cerebellum (Savic et al. 2000; Suzuki et al. 2001; Kjelvik et al. 2012), the right temporal as well as the right parietal cortex (Savic et al. 2000; Suzuki et al. 2001). Clinical studies seem to support these observations. For instance, focal cerebellar lesions caused impairment of odor threshold and odor identification (Mainland et al. 2005; Zobel et al. 2010). In a nutshell, several overlapping neural circuits are involved in the processing of higher olfactory functions. CD may be a network disorder (Prudente et al. 2014; Quartarone and Hallett 2013; Shakkottai et al. 2017) and our findings of olfactory decline in CD support this idea rather than pointing to a single site of pathology in CD.

Standardized gustatory testing revealed diminished perception of taste in CD subjects compared to healthy controls. The reason for this is not completely clear. The participation of the sensorimotor cortex in the pathophysiology of CD (Prudente et al. 2014; Quartarone and Hallett 2013; Shakkottai et al. 2017) and the perception of the tactile components of taste (Mascioli et al. 2015; Wistehube et al. 2018) may play a role in gustatory decline in CD. Olfactory decline can lead to lower gustatory functioning (Landis et al. 2010; Rolls et al. 2005) and may have contributed to the present findings as well.

Similar to findings of studies on the chemical sense in aging, the present data analysis suggests that age may a predictor for lower performance in the olfactory tests in CD (Doty 2018; Hummel et al. 2007; Mueller et al. 2003; Murphy et al. 2002). Also, cognitive functioning may play a role, as lower MoCA scores predicted lower performance with the composite taste score in CD subjects. Interestingly, the TWSTRS—part 3, which assesses the severity of pain in CD, predicted lower performance in the odor threshold test. Confirmation of present findings through additional studies involving more CD subject is warranted.

This study has limitations. Although being the first study on taste in CD and the second largest study on olfaction in CD (Marek et al. 2018) involving a sizable number of subjects to address the primary aim of this study, the number of CD subjects assessed may have been too small to analyze factors with small to moderate impact on the chemical senses. Second, tobacco smokers were allowed into this study. This was done to better reflect the cohort of subjects with CD. However, tobacco smoking (especially ≥ 20 cigarettes per day) negatively impacts the chemical senses (Vennemann et al. 2008). To control for this confounding factor, the same number of healthy subjects with the same smoking burden were included. Our analyses suggest that tobacco smoking may not be the main factor for decline of the chemical senses in CD and confirms findings of an independent study on the sense of smell in CD (Marek et al. 2018). A third limitation is that the assessment of the study participants was done unblinded, which could have caused bias. However, established and validated psychophysical assessment tools were applied by trained examiners for the examination of the chemical senses (Hummel et al. 1997; Mueller et al. 2003). Finally, besides the BSI, which is a widely used, reliable and validated questionnaire (Tarescavage and Ben-Porath 2014), the Structured Clinical Interview for DSM-III-R (SCID) (Spitzer et al. 1992) may be added in future studies on the chemical senses to more precisely quantify psychiatric alteration in CD.

In conclusion, findings propose that CD is associated with diminished olfactory and gustatory functioning. The assessment of a larger number of CD subjects may confirm present findings and to see if an impairment of the chemical senses is a potential endophenotype of CD. Future studies may also assess whether therapeutic intervention such as deep brain stimulation targeting the basal ganglia or BTX impacts the chemical senses in CD.
