# Correction to: Smell and taste in cervical dystonia

**DOI:** 10.1007/s00702-021-02340-0

**Published:** 2021-05-06

**Authors:** Thorsten Herr, Thomas Hummel, Marcus Vollmer, Carsten Willert, Birgitt Veit, Julie Gamain, Robert Fleischmann, Bernhard Lehnert, Jan-Uwe Mueller, Andrea Stenner, Martin Kronenbuerger

**Affiliations:** 1 Department of Neurology, University Medicine Greifswald, Ferdinand-Sauerbruch-Strasse, 17475 Greifswald, Germany; 2 Department of Otorhinolaryngology, TU Dresden, Dresden, Germany; 3 Institute of Bioinformatics, University Medicine Greifswald, Greifswald, Germany; 4 Neurology Group Practice, Stralsund, Germany; 5 Neurology Group Practice, Neubrandenburg, Germany; 6 Department of Otorhinolaryngology, University Medicine Greifswald, Greifswald, Germany; 7 Department of Neurosurgery, University Medicine Greifswald, Greifswald, Germany; 8 Outpatient Department of Neurology, Paracelsus Clinic Zwickau, Zwickau, Germany; 9 Department of Neurology, Johns Hopkins University, Baltimore, MD USA

## Correction to: Journal of Neural Transmission (2020) 127:347–354 10.1007/s00702-020-02156-4

The article Smell and taste in cervical dystonia, written by Thorsten Herr, Thomas Hummel, Marcus Vollmer, Carsten Willert, Birgitt Veit, Julie Gamain, Robert Fleischmann, Bernhard Lehnert, Jan‑Uwe Mueller, Andrea Stenner and Martin Kronenbuerger, was originally published Online First without Open Access. After publication in volume 127, issue 3, pages 347–354 the author decided to opt for Open Choice and to make the article an Open Access publication. Therefore, the copyright of the article has been changed to © The Author(s) 2021 and this article is licensed under a Creative Commons Attribution 4.0 International License, which permits use, sharing, adaptation, distribution and reproduction in any medium or format, as long as you give appropriate credit to the original author(s) and the source, provide a link to the Creative Commons licence, and indicate if changes were made. The images or other third party material in this article are included in the article’s Creative Commons licence, unless indicated otherwise in a credit line to the material. If material is not included in the article’s Creative Commons licence and your intended use is not permitted by statutory regulation or exceeds the permitted use, you will need to obtain permission directly from the copyright holder. To view a copy of this licence, visit http://creativecommons.org/licenses/by/4.0/.

The original article has been corrected.

